# The New Method of Inducing Sleep without Drugs

**Published:** 1900-09

**Authors:** J. B. Learned

**Affiliations:** Northampton, Mass.


### Domestic Correspondence.

### THE NEW METHOD OF INDUCING SLEEP WITHOUT DRUGS.—ONE HUNDRED DOLLAR PRIZE.

To the Editor:

Sir,—The new method of inducing sleep without drugs consists in bringing under will-power the functions of organic life immediately on retiring. The organs of respiration and circulation respond to our bidding. Certain other groups of muscle, by contraction and relaxation, are made accessory in directing the arterial and vital currents away from the gray matter of the brain. As a result, automatic thinking, which is the immediate cause of sleep's delay in the case of the average brain-working business man, is absolutely shut out, and normal sleep is inevitable.

To give the technique in full would require more space than can be given here. Suffice it, that mental and physical conditions, positions, and changes, extemporized and controlled by will-power in the horizontal position, with suitable temperature and ventilation of body-surface as well as lungs, is the sheet-anchor of "The New Method."

Believing that the medical profession has power to turn the attention of suffering humanity from the mysterious chimerical and damaging drug agents they now depend upon, and that it is our duty to shed light rather than darkness, I offer a prize of one hundred dollars for an essay which shall describe any method of extemporizing sleep immediately on retiring for the brain-working classes, that will equal or surpass the "New Method" above referred to.

Time allotted for preparing essays, from June to December of the present year. Length of essay not to exceed five thousand words; to be in print or type-written, without the name of the writer. Judges of the merits of essays shall be representative men of scientific medicine. Time for awarding prize, January 1, 1901. Results will be announced in this journal.

J. B. Learned.

Northampton, Mass.

